# Peri-Operative Impact of Cannabis Use in Conjunction With Elective Primary Breast Augmentation in a Private Practice

**DOI:** 10.7759/cureus.57196

**Published:** 2024-03-29

**Authors:** Shayna R Mehta, Adrienne Stolfi, Michael R Johnson, Usha Rajagopal, Spencer R Anderson

**Affiliations:** 1 Plastic and Reconstructive Surgery, Wright State Boonshoft School of Medicine, Dayton, USA; 2 Pediatrics, Wright State Boonshoft School of Medicine, Dayton, USA; 3 Plastic Surgery, San Francisco Plastic Surgery and Laser Center, San Francisco, USA

**Keywords:** effects of cannabis use, post-operative complication, recreational cannabis, implant-based breast augmentation, cannabis use

## Abstract

Background

The consumption of recreational and medicinal cannabis in the United States continues to increase. Understanding the effects of cannabis in patients undergoing elective primary breast augmentation (EPBA) is of paramount importance with the expanding rates of reported cannabis consumption.

Objectives

This study aims to analyze the peri-operative impact of cannabis use in conjunction with EPBA in a single-surgeon practice in San Francisco, California.

Methods

A retrospective chart review was performed of 134 adult female patients undergoing EPBA from August 2018 to January 2022 within a single-surgeon practice plastic surgery office. Cannabis use was self-reported as current use or former use. Cohorts were grouped as cannabis users and cannabis non-users.

Results

Of the 134 patient charts identified for analysis, 58 (43.3%) reported cannabis use. Cannabis users were significantly younger than cannabis non-users (26.8 years versus 31.5 years, *P*<0.001). No significant differences were found between groups among intra-operative blood loss, post-operative complication rates, post-operative narcotic use, or intra-operative anesthetic requirements. The incidence of adverse events, including wound breakdown, skin necrosis, and capsular contracture requiring reoperation, did not differ significantly between cannabis users and cannabis non-user groups. Ninety-six percent of patients had their implants placed subpectorally, and all procedures were done using a Keller funnel. Eighty-three percent of patients had Sientra implants, and 96% of all implants were silicone gel implants. All procedures were done under general anesthesia. Patients were followed for up to two years.

Discussion

This review found no significant differences in peri-operative and post-operative outcomes between cannabis users and cannabis non-users.

## Introduction

The consumption of recreational and medicinal cannabis in the United States has grown rapidly, with 34.5% of people aged 18 to 25 years reporting cannabis use in the past year [1]. In 1996, California was the first state to approve cannabis for medicinal use [2]. As of April 2023, 38 states, three territories, and the District of Columbia allow the medical use of cannabis or cannabis-based products. Additionally, 22 states, two territories, and the District of Columbia have recently passed laws to regulate recreational cannabis consumption [3].

Cannabis sativa, a versatile plant used for fiber, food, and medicine, has a long history of medicinal applications dating back to 2700 B.C. in China. Over 100 identified cannabinoids in the plant exert pharmacological effects by interacting with receptors in the endocannabinoid system [4]. Currently, medical applications of cannabis include the treatment of chronic pain, chemotherapy-related nausea and vomiting, anorexia due to HIV/AIDS, multiple sclerosis-induced spasticity, anxiety, and sleep disorders [5].

The pharmacokinetics of cannabinoids vary depending on the route of administration and the dose and frequency of cannabis consumption. Cannabis products are available in various dosage forms and routes of administration, including inhalation, oral, sublingual, and topical. Inhalation products include smoked flowers and vaporized concentrates. Orally ingested cannabis dosage forms include oils, oral solutions, capsules, tablets, and food products [6].

Understanding the effects of cannabis in the peri-operative setting is increasingly important with expanding consumption rates. There is limited research concerning the peri-operative impact of cannabis use in plastic surgery, specifically elective primary breast augmentation (EPBA) [7,8]. Cannabis use continues to grow with wider societal acceptance and expanding legalization [9,10]. With cosmetic plastic surgery clinics experiencing an increasing trend of cannabis users presenting for surgical consultations, a better understanding of the potential effects of cannabis use in an elective setting is warranted. This study aims to analyze the impact of cannabis use in conjunction with EPBA in a single-surgeon private practice in San Francisco, California.

## Materials and methods

A retrospective chart review was performed on 134 adult female patients undergoing EPBA from August 2018 to January 2022 within a single-surgeon plastic surgery office in San Francisco, California. Patients were identified via office records based on office-specific identifiers. Approval for this investigation was granted through the Institutional Review Board.

The study's inclusion criteria and review parameters included female patients, 18 years of age or older, undergoing isolated EPBA with either silicone or saline implants, followed by at least one postprocedural follow-up. Patients were followed for at least two years. Exclusion criteria included tobacco use, illicit drug use (i.e., cocaine, LSD, etc.), benzodiazepine use, or an additional procedure at the time of surgery. Twenty-eight patients were excluded due to reporting concurrent tobacco use or other excluded drug use.

Variables analyzed included age, body mass index (BMI), cannabis use status, comorbidities, peri-operative anesthetics, duration of surgery, and post-operative complications. Cannabis use status was self-reported as current use. The type of cannabis or cannabinoid product, dosage form, and route of administration were not available for inclusion in the study. Two cohorts were established as cannabis non-users and cannabis users.

Statistical analysis

Categorical variables were described with frequency (percent of non-missing values) and continuous variables with mean (standard deviation, SD). Comparisons of categorical variables between cannabis users and cannabis non-users were made with chi-square or Fisher’s exact tests. Right and left implant sizes were normally distributed and were compared between groups with two-sample t-tests. All other continuous variables were right-skewed and compared with Mann-Whitney U tests. For implant sizes, analysis of covariance was used to determine the associations between the user group and implant sizes after adjusting for age. The data were analyzed with IBM SPSS for Windows version 29.0 (IBM Corporation, Armonk, NY). P-values less than 0.05 were considered statistically significant.

## Results

One hundred and thirty-four female patients were identified based on the criteria. Fifty-eight patients (43.3%) self-reported isolated cannabis use. The mean (SD) age of all patients was 29.5 (7.0) years; cannabis users were younger at 26.8 years versus 31.5 years for the cannabis non-user group (P<0.001). BMI was 21.5 (2.8) kg/m2, with no differences between the groups (Table 1). Forty-three percent of patients were Asian, 26.5% were White, 19.7% were Hispanic, 3.0% were Black, and 7.6% were other/multiple races, with no significant differences in race between the cannabis user and cannabis non-user groups (Table 1).

**Table 1:** Descriptive statistics SD, standard deviation.

Demographics	Cannabis non-users (n=76)	Cannabis users (n=58)	All patients (n=134)	P-value
Age (years), mean (SD)	31.5 (7.4)	26.8 (5.4)	29.5 (7.0)	<0.001
BMI (kg/m^2^), mean (SD)	21.6 (3.0)	21.2 (2.5)	21.5 (2.8)	0.423
Race (n=132), n (%)				
White	18 (24.3)	17 (29.3)	35 (26.5)	0.147
Black	3 (4.1)	1 (1.7)	4 (3.0)	
Asian	31 (41.9)	26 (44.8)	57 (43.2)	
Hispanic	19 (25.7)	7 (12.1)	26 (19.7)	
Other/multiple	3 (4.1)	7 (12.1)	10 (7.6)	
Currently taking an oral contraceptive or hormone replacement (n=130), n (%)	21 (28.4)	23 (41.1)	44 (33.8)	0.13
Caprini risk scale, n (%)				
Very low/Low (0-2)	34 (44.7)	28 (48.3)	62 (46.3)	0.420
Moderate (3-4)	40 (52.6)	26 (44.8)	66 (49.3)	
High (≥5)	2 (2.6)	4 (6.9)	6 (4.5)	

There were no significant differences in post-operative opioid use or peri-operative or intra-operative anesthetic requirements. Pre-operative medications administered included acetaminophen (33.6% of patients), cefazolin (92.5%), gabapentin (34.3%), gentamycin (4.5%), and vancomycin (0.7%). Intra-operative medications administered included fentanyl (93.3%), midazolam (95.5%), ondansetron (85.8%), and propofol (97.8%). Immediate post-operative medications administered included fentanyl (0.7%) and promethazine (1.5%). Post-operative medications at discharge included acetaminophen with hydrocodone (87.3%) and gabapentin (26.1%).

There were no differences between the groups in duration of surgery, incidence of intra-operative blood loss, or post-operative complication rate (Table 2). Additionally, rates of adverse events such as wound breakdown, skin necrosis, hematoma, and capsular contracture requiring re-operation were equal between the groups (Table 2). Interestingly, cannabis non-users elected to have larger breast implants (right implant P=0.004, left implant P=0.002, Table 2). These differences remained significant after adjusting for differences in age between the user and non-user groups with analysis of covariance (P=0.017, right implant; P=0.009, left implant). Ninety-six percent of patients' implants were sub-pectoral, and all procedures were done using a Keller funnel. Eighty-three percent of patients had Sientra implants, and 96% of all implants were silicone gel implants. All procedures were done under general anesthesia. Patients were followed for up to two years after surgery.

**Table 2:** Intra-operative statistics NA: not applicable; SD: standard deviation.

Intra-operative statistics	Cannabis non-users (n=76)	Cannabis users (n=58)	P-value
Propofol dose (mg) (n=131), mean (SD)	185 (48)	187 (34)	0.468
Implant size (cc), right, mean (SD)	395 (83)	355 (75)	0.004
Implant size, (cc), left, mean (SD)	394 (84)	349 (77)	0.002
Minutes from time in the room to incision, mean (SD)	18 (8)	17 (7)	0.977
Minutes from incision to close, mean (SD)	56 (10)	54 (11)	0.233
Minutes from time in room to time out of room, mean (SD)	80 (13)	78 (16)	0.134
Minutes from close to time out of room, mean (SD)	7 (5)	7 (4)	0.753
Minutes from time out of room to discharge, mean (SD)	51 (18)	54 (22)	0.808
Intra-operative excess bleeding (n=132), n (%)	2 (2.7)	1 (1.8)	1
Post-operative reoperation (n=130), n (%)	5 (6.8)	6 (10.7)	0.529
Type of post-operative re-operation (n=11), n (%)			
Implant malposition	1 (20.0)	3 (50.0)	0.545
Desire size change	4 (80.0)	3 (50.0)	
Wound breakdown/wound care at closure site, (n (%)	2 (2.7)	0 (0.0)	0.506
Skin necrosis, n (%)	1 (1.3)	1 (1.7)	1
Hematoma (n, %)	0 (0.0)	0 (0.0)	NA
Capsular contracture requiring reoperation, n (%)	0 (0.0)	1 (1.8)	0.432

## Discussion

In this retrospective chart review, no statistically significant differences were found in pre-operative or intra-operative anesthetic use, duration of surgery, post-operative narcotic use, or adverse events for cannabis users compared to cannabis non-users.

Cannabis users and non-users had similar peri-operative and post-operative outcomes. There was no significant difference between groups in intra-operative blood loss or post-operative complications. The incidence of adverse events, including wound breakdown, skin necrosis, and capsular contracture requiring re-operation, did not differ significantly between the cannabis user and non-user groups. Previous research demonstrates an association between cannabis abuse, formally known as cannabis use disorder, and increased peri-operative complications, such as thromboembolism and respiratory complications. Post-operative complications of cannabis use disorder include sepsis or septicemia, neurological complications such as anoxic brain injury and stroke, increased lengths of stay, and higher hospitalization charges [11]. Chronic cannabis use is also associated with similar pulmonary complications as tobacco inhalation, including airway inflammation. Furthermore, the use of cannabis within 72 hours of general anesthesia is advised against, as it can increase cardiac workload, may cause airway obstruction, may necessitate higher anesthetic dosages in the laryngeal airways, and increases the risk of stroke and myocardial infarctions [12].

The data compiled from this review did not indicate whether patients were chronic cannabis users or diagnosed with cannabis use disorder. However, the findings did not demonstrate significant differences between groups with regard to peri-operative complications, which indicates that cannabis use disorder may not have been present for the patients surveyed. This observation also suggests that the frequency of cannabis use and pathological responses to cannabis may increase susceptibility to peri-, intra-, and post-operative complications.

Cannabis use continues to be an increasing trend in the United States. An analysis of data from over 16,000 women ages 18 to 44 in 12 US states found that 9.9% reported current cannabis use [13]. Although a common occurrence, cannabis use is not without significant risk in certain patient populations. A prior retrospective study of 27,306 patients found that post-operative myocardial infarction rates were increased in patients with cannabis use disorder who underwent orthopedic, cardiac, obstetric, and general surgery-based procedures. However, there was no difference in the rates of peri-operative morbidity, mortality, length of stay, or costs [14]. Concerning pain management, prior studies have shown that chronic cannabis consumption may worsen post-operative pain, increase post-operative opioid use, and precipitate the development of post-operative hyperalgesia [15]. However, in our investigation, peri-operative anesthetic and post-operative narcotic requirements did not differ significantly between the cannabis user and non-user groups.

The American Society of Regional Anesthesia and Pain Medicine guidelines recommend universal pre-operative screening of patients for cannabinoid consumption, including the type of product consumed, time of last consumption, route of administration, amount, and frequency of use. The guidelines recommend delaying elective surgery for a minimum of two hours after a patient has smoked cannabis to minimize cardiovascular risks and ensure patients are able to provide informed consent [15].

Limitations

While this study provided important information on the impact of pre-operative cannabis consumption in EPBA, it is not without noted limitations to discuss. One of the main limitations of the retrospective nature of this review due to self-reported cannabis use is potential recall bias, as it may have been difficult for the patients to accurately remember the frequency or dose of cannabis used in the past. Another important limitation was the lack of reported specifics on the timing, dose, and form of cannabis consumed prior to surgery. Additional limitations that introduced possible inaccuracies and biases included incomplete data on the frequency and type of cannabis used regularly, uncontrolled confounders, such as diet or exercise, and whether cannabis was used for medical purposes or solely as a recreational drug. Furthermore, dose, dosage form, and route of administration could cause distinct effects, such as potential differences in smoking versus other dosage forms on pulmonary function. The route of cannabis administration, which can be oral, smoked, or vaporized, alters cannabis pharmacokinetics as well as its subjective and cardiorespiratory effects. These are important factors to take into consideration for future research. In addition, cannabis inhalation demonstrates faster peak blood plasma levels and higher bioavailability compared to the consumption of cannabis edibles or oil. Therefore, the comparison of patients who prefer inhalation rather than oral consumption may provide important data regarding the effect of cannabis use on surgical outcomes.

The absence of cannabis-specific patient information such as product type, frequency, and consumption route (inhalation vs. oral ingestion) limited the ability to directly ascertain whether cannabis use alone had a direct impact on peri- and post-operative outcomes, warranting further study with comprehensive data for cannabis users. Another limitation to consider is the lack of generalizability of the results due to the use of a single site with a single provider and the small sample size of 134 patients.

Despite expanding societal acceptance, especially in California, stigmatization of cannabis use remains a barrier to patient self-reporting. Surveys of cannabis consumers have shown that patients experience high rates of hesitation in disclosing cannabis use to healthcare providers, including fear of judgment, loss of employment, and legal repercussions [16]. Therefore, the limited data obtained from self-reports may be attributed to the ongoing stigmatization of cannabis use as opposed to recall bias.

## Conclusions

In conclusion, no statistically significant difference in overall peri-operative outcomes was identified when comparing cannabis and non-cannabis users undergoing EBPA in our single-surgeon, private practice study. Cannabis use continues to be a growing trend with the increasing adoption of both medicinal and recreational use among patients presenting for plastic surgery consultation. Ongoing evaluation into the possible impact of cannabis use on surgical outcomes is an important area warranting additional investigation in this specific patient population. Furthermore, it is anticipated that as the stigmatization of cannabis use eases due to the adoption of recreational and medicinal use, patients may be more forthcoming with regard to the frequency, dose, and type of cannabis product consumed. Improved disclosure of cannabis use may further elucidate the potential impact on surgical outcomes. This review emphasizes the need for future studies involving larger populations and detailed information pertaining to cannabis use status to improve patient-physician awareness.
